# Evaluation of CEMIP in diagnosis of pancreatic carcinoma in comparison with other traditional markers

**DOI:** 10.1038/s41598-025-07911-9

**Published:** 2025-07-03

**Authors:** Randa Ahmed El Zohne, Ahmad Kamel Mostafa Abo Zaid, Omnia Abd El Moneim, Ahmed Mohamed Soliman, Dina Mohamed Safwat, Soad A. Eltokhy

**Affiliations:** 1 Department of Clinical Pathology, Faculty of Medicine, Assiut University, Assiut, 17515 Egypt; 2 Department of General Surgery-Faculty of Medicine, Assiut University, Assiut, Egypt; 3 Clinical Pathology Department, National Cancer Institute, Cairo University, Cairo, Egypt

**Keywords:** Pancreatic cancer, Cell migration inducing protein, Markers, Accuracy, Pancreatic cancer, Cancer screening, Gastrointestinal cancer, Gastrointestinal diseases

## Abstract

Screening and early diagnosis of pancreatic cancer (PC) are crucial for improving its prognosis. In the current study, we aimed to evaluate the clinical utility of serum cell migration inducing protein in pancreatic cancer patients (CEMIP). This study was conducted on 50 newly diagnosed pancreatic cancer patients, aged from 49 to 77 years. The study also included 20 patients with benign intestinal diseases, and 20 apparently healthy individuals who were selected as a control group for comparison. Practical work was carried out at Clinical Pathology Department, Assiut University Hospital. All groups were subjected to thorough history taking and clinical evaluation. Radiological data and laboratory tests in addition evaluation level of carcinoembryonic antigen (CEA), cancer antigen 19 − 9 (CA19-9) and CEMIP were recorded. Pancreatic cancer group had significantly higher CEA, CA19-9 and CEMIP compared to both benign GIT diseases and control group, with (P-value < 0.001) for each. Late pancreatic cancer group had significantly higher CEA, CA19-9 and CEMIP compared to early pancreatic cancer with (P-value = 0.01). For diagnosis of PC, CEMIP was 95% sensitive and 84% specific, with AUC of 0.86 while CEA was 80% sensitive and 65% specific, with AUC of 0.80 and that of serum CA 19 − 9 was 58% sensitive and 69% specific, with AUC of 0.80. For diagnosis of early PC, CEMIP was 90% sensitive and 83% specific, with AUC of 0.72. These results are better than that of serum CEA, which was 75% sensitive and 60% specific, with AUC of 0.52 and that of serum CA 19 − 9, which was 60% sensitive and 58% specific, with AUC of 0.56. Serum CEMIP may serve as non-invasive biomarkers for diagnosis of pancreatic cancer patients in comparison to other conventional biomarkers.

## Introduction

The global incidence of pancreatic cancer (PC) has been steadily rising, and it continues to exhibit the highest mortality rate and the lowest survival rate among all malignancies^1^. Carbohydrate antigen 19 − 9 (CA19-9) and carcinoembryonic antigen (CEA) are widely utilized in the clinical diagnosis of gastrointestinal malignancies; however, their diagnostic accuracy remains limited^2–5^.

Cell migration inducing protein (CEMIP) is overexpressed in various cancers, and is associated with an aggressive phenotype. Notably, it enhances the motility of colon cancer cells and is therefore called the cell migration-inducing protein (CEMIP)^6–8^.

CEMIP has been found to be overexpressed in various malignancies, including breast, colorectal, hepatic, gastric, pancreatic, prostate, and ovarian cancers. Increasing evidence suggests that its overexpression is associated with enhanced metastatic potential and poor prognosis in several human tumors^9–11^.

To our knowledge, till this date, there was only one study that discussed serum level of CEMIP among patients with PC. This study displayed that CEMIP showed a diagnostic rate of 86.1% (68/79) in pancreatic cancer. Combined CA19-9 with CEMIP had better diagnostic accuracy in prediction the pancreatic cancer^12^.

Based on confirmed role of CEMIP in many malignant lesions, our rationale in the current study was to determine applicability of CEMIP in early prediction of pancreatic cancer and if it could be used as a promising biomarker in this area.

The current study aimed to estimate the level of CEMIP, CA19-9 and CEA in pancreatic cancer patients and evaluate the clinical utility of serum CEMIP, CA19-9 and CEA in diagnosis of pancreatic cancer patients.

## Patients and methods

### Study setting and design

A case control study was conducted at General Surgery Department and Clinical Pathology Department of Assiut University Hospitals. It was performed in the period between January 2021 till 1st January 2023.

### Selection criteria

#### Inclusion criteria

Patients with pancreatic cancer based on clinical, radiological and histopathological evaluation were eligible to the study.

#### Exclusion criteria

- pancreatic surgery.
- malignancy other than pancreatic cancer.
- refusal to participate in the study.

### Participants

The study recruited the follow groups;

- First group included 50 patients with pancreatic cancer based on clinical, radiological and histopathological evaluation (PC group).
- Second group included 20 patients with benign diseases (benign group).
- Third group included 20 healthy subjects as control group.

### Ethical consideration

The study was conducted according to the principles of the Declaration of Helsinki and was approved by the Hospital’s Ethics Committee purpose of the study was explained to all participants.

A written informed consent was obtained. The study was approved by Assiut Faculty of Medicine, Institutional Review Board (IRB No. 17101011, 2021). The study was explained to all patients and only patients who were signing an informed consent were participated in study. This study was registered on clinicaltrials.gov with identifier: (NCT04101110).

## Methodology

### Baseline clinical evaluation

All patients were subjected to thorough history taking and clinical evaluation. Clinical data of patients were recorded.

### Staging of patients with pancreatic cancer

Early-stage PC were defined as cases with stage I and stage II (invasive carcinoma with tumor diameter of < 20 mm confined within the pancreas with the absence of regional lymph node metastasis. While late-stage PC included stages III-IV where stage III: Wider spread and stage IV: Confirmed spread^13^.

#### Laboratory data among the studied groups

Baseline laboratory tests were done in all groups included complete blood count, coagulation profile, liver function tests, kidney function tests, amylase and lipase.

CEA and CA19-9 measurement was done the ADVIA Centaur CEA assay is a two-site sandwich immunoassay using direct chemiluminometric technology, which uses constant amounts of two antibodies.

### Measurement of CEMIP

The levels of CEMIP were measured using the enzyme-linked immunosorbent assay (ELISA) method. Secreted CEMIP protein was detected in blood samples obtained from patients with pancreatic cancer and individuals without cancer. A CEMIP ELISA kit (SER965Hu) was purchased from USCN Life Science, Inc. (Wuhan, China) and the procedure was performed according to the manufacturer’s instructions. The detection range for the ELISA kit used was 0.156–10 ng/ml.

### Statistical analysis

Data was collected and analyzed by using SPSS (Statistical Package for the Social Science, version 20, IBM, and Armonk, New York). The Shapiro test was used to determine compliance of the data to normal distribution. Quantitative data with normal distribution are expressed as mean ± standard deviation (SD) and compared with ANOVA test with post-hoc analysis. Quantitative data with abnormal distribution expressed as median (minimum-maximum) and compared by Kruskal Wallis and Mann-Whitney U test was used.

Nominal data are given as number (n) and percentage (%). Chi^2^ test was implemented on such data. Accuracy of different biomarkers in diagnosis of PC and late stage of PC was determined by receiver operator characteristics (ROC) curve. To compare the diagnostic performance of the different biomarkers, the DeLong test was used to evaluate the statistical significance of the difference between the areas under the ROC curves (AUCs). Spearman coefficient correlation was used to determine correlation between CEMIP with other variables. Level of confidence was kept at 95% and hence, P value was considered significant if < 0.05.

## Results

### Baseline data of the studied groups (Table 1)

There were no statistically significant differences between the studied groups.

**Table 1:** Baseline data of the studied groups.

	PC group (*n* = 50)	Benign group (*n* = 20)	Control group (*n* = 20)	*P* value	Subgroup analysis		
					P1	P2	P3
Age (years) Range	64.18 ± 10.67 49–77	60.98 ± 5.98 39–68	59.34 ± 12.45 38–65	0.98	0.08	0.12	0.77
Sex				0.57	0.09	0.11	0.40
Male	27 (54%)	13 (65%)	12 (60%)				
Female	23 (46%)	7 (35%)	8 (40%)				
Family history of PC	3 (6%)	1 (5%)	0	0.54	0.78	0.08	0.07
Smoking	14 (28%)	6 (30%)	7 (35%)	0.18	0.06	0.49	0.10
DM	8 (16%)	3 (15%)	0	0.45	0.51	0.09	0.34
HTN	5 (10%)	2 (10%)	0	0.19	0.08	0.40	0.90
IHD	1 (2%)	1 (5%)	0	0.08	0.26	0.19	0.54
CKD	3 (6%)	1 (5%)	0	0.51	0.10	0.45	0.50
Other comorbidities	3 (6%)	1 (5%)	0	0.90	0.09	0.82	0.79

Data expressed as frequency (percentage), mean (SD). *P* value was significant if < 0.05. PC: pancreatic cancer; DM: diabetes mellitus; HTN: hypertension; IHD: ischemic heart disease; CKD: chronic kidney disease. *P* value compares between the studied groups; *P*1 compares between PC vs. Benign groups; *P*2 compares between PC vs. control groups; *P*3 compares between Benign and control groups.

### Clinical manifestations and diagnosis of studied patients (Table 2)

Table 2 summarize clinical presentation of studied groups.

**Table 2:** Clinical manifestations among the studied patients.

	PC group (*n* = 50)	Benign group (*n* = 20)	*P* value
Clinical presentation			
Dark red urine	50 (100%)	13 (65%)	< 0.001
Jaundice	50 (100%)	13 (65%)	< 0.001
Clay stool	50 (100%)	13 (65%)	< 0.001
Abdominal pain	17 (34%)	11 (55%)	0.03
Itching	15 (30%)	2 (10%)	0.02
Fever	5 (10%)	10 (50%)	< 0.001
Loss of weight	12 (24%)	0	< 0.001
Diagnosis			
Early PC	29 (58%)	0	
Late PC	21 (42%)	0	
COJ	0	13 (65%)	
Gall stones	0	5 (25%)	
Chronic pancreatitis	0	2 (10%)	

Data expressed as frequency (percentage). *P* value was significant if < 0.05. PC: pancreatic cancer; COJ: calcular obstructive jaundice.

### Radiological evaluation of patients with pancreatic cancer (Table 3)

Table 3 summaries radiological data (abdominal ultrasound and computed tomography) of pancreatic cancer.

**Table 3:** Radiological evaluation of patients with pancreatic cancer.

	*N* = 50
Maximum diameter (cm) Range (cm)	5.10 ± 1.31 2.30–7.60
Vascular invasion	21 (42%)
Abdominal lymphadenopathy	10 (20%)
Ascites	5 (10%)

Data expressed as frequency (percentage), mean (SD), range.

### Serum biomarkers among the studied groups (Table 4)

PC group had significantly higher CA19-9, CEA and CEMIP in comparison to other groups. Levels of these biomarkers were comparable in the benign and control groups.

**Table 4:** Serum biomarkers of the studied groups.

	PC group (*n* = 50)	Benign group (*n* = 20)	Control group (*n* = 20)	*P* value	Subgroup analysis		
					P1	P2	P3
CA19-9 (u/l) Range	63.27 ± 49.92 12–221	16.55 ± 10.61 3.11-37	10.29 ± 9.09 1–30	**< 0.001**	**< 0.001**	**< 0.001**	0.53
CEA (ng/ml) Range	26.04 ± 30.13 3.40–123	6.09 ± 3.17 1.09-10	1.55 ± 0.37 1-2.20	**< 0.001**	**< 0.001**	**< 0.001**	0.06
CEMIP (ng/ml) Range	3.55 ± 2.08 0.76–8.77	1.60 ± 0.89 0.23–3.98	1.31 ± 0.72 0.23–2.95	**< 0.001**	**< 0.001**	**< 0.001**	0.13

Data expressed as mean (SD). P value was significant, if < 0.05. PC: pancreatic cancer; CEA: carcinoembryonic antigen; CA19-9: cancer antigen 19 − 9; CEMIP: cell migration inducing protein.

P value compares between the studied groups; P1 compares between PC vs. Benign groups; P2 compares between PC vs. control groups; P3 compares between Benign and control groups.

### Serum biomarkers in PC based on its stage (Table 5)

CEMIP, CEA and CA19-9 were significantly higher among those with late PC in comparison to those with early PC.

**Table 5:** Serum biomarkers in patients with PC based on its stage.

	Early PC (*n* = 29)	Late PC (*n* = 21)	*P* value
CA19-9 (u/l) Range	54.87 ± 39.54 12-190.67	74.88 ± 60.61 12.98–221	**0.01**
CEA (ng/ml) Range	17.54 ± 22.13 1.09–17.20	37.87 ± 35.91 10–123	**0.01**
CEMIP (ng/ml) Range	2.69 ± 1.11 0.76–5.33	4.73 ± 2.52 1.96–8.77	**0.01**

Data expressed as mean (SD). *P* value was significant if < 0.05. PC: pancreatic cancer; CEA: carcinoembryonic antigen; CA19-9: cancer antigen 19 − 9; CEMIP: cell migration inducing protein.

### Correlation of CEMIP with other variables (Table 6)

It was found that CEMIP had positive significant correlations with CA19-9 (*r* = 0.37, *p* < = 0.01) and CEA (*p* = 0.65, *p* = 0.001) in patients with pancreatic cancer.

**Table 6:** Correlation of CEMIP with other variables in each group.

	PC group	Benign group	Control group
Age (years)	0.04 (0.98)	0.11 (0.87)	0.21 (0.40)
PC diameter (cm)	0.10 (0.45)		
AST (u/l)	− 0.05 (0.62)	− 0.08 (0.47)	0.09 (0.55)
ALT (u/l)	− 0.19 (0.09)	− 0.09 (0.39)	0.12 (0.26)
Bilirubin (mg/dl)	− 0.06 (0.56)	− 0.02 (0.84)	0.03 (0.78)
Direct bilirubin (mg/dl)	0.11 (0.56)	0.13 (0.49)	0.01 (0.49)
Indirect bilirubin (mg/dl)	0.09 (0.20)	− 0.10 (0.51)	0.09 (0.18)
Albumin (g/dl)	− 0.1 (0.93)	0.03 (0.76)	− 0.04 (0.69)
Total proteins (g/dl)	0.10 (0.19)	0.09 (0.19)	0.18 (0.20)
ALP (u/l)	0.07 (0.54)	− 0.03 (0.74)	− 0.02 (0.80)
GGT (u/l)	0.08 (0.12)	− 0.11 (0.89)	0.01 (0.39)
FBS (mol/ll)	0.21 (0.56)	− 0.07 (0.09)	− 0.04 (0.55)
Urea	0.10 (0.19)	0.21 (0.61)	− 0.11 (0.10)
Creatinine (mmol/l)	0.05 (0.65)	0.14 (0.20)	− 0.08 (0.43)
Sodium (mmol/l)	0.12 (0.27)	0.06 (0.55)	0.05 (0.60)
Potassium (mmol/l)	0.04 (0.70)	0.07 (0.51)	− 0.15 (0.17)
INR	0.14 (0.21)	0.24 (0.40)	− 0.03 (0.79)
PT (second)	− 0.13 (0.23)	0.01 (0.99)	0.17 (0.12)
PC (%)	0.21 (0.06)	0.06 (0.58)	0.15 (0.16)
Leucocytes (10^3^/ul)	0.21 (0.06)	0.11 (0.33)	0.06 (0.56)
Hemoglobin (g/dl)	0.04 (0.69)	0.01 (0.90)	− 0.01 (0.67)
Platelets (10^3^/ul)	− 0.01 (0.87)	− 0.21 (0.06)	− 0.04 (0.69)
CA19-9 (u/l)	**0.37 (0.01)**	0.018(0.17)	0.13 (0.65)
CEA (ng/ml)	**0.65 (0.001)**	0.09 (0.92)	0.12 (0.20)

Data expressed as *r* value (*p* value). *P* value was significant if < 0.05. PC: pancreatic cancer; CEMIP: cell migration inducing protein; LFT: liver function test; KFT: kidney function test; AST: aspartate transaminase; ALT: alanine transaminase; ALP: alkaline phosphatase; GGT: gamma glutamyl transpeptidase; FBS: fasting blood sugar; INR: international randomized ratio; PT: prothrombin time; CEA: carcinoembryonic antigen; CA19-9: cancer antigen 19 − 9.

### Accuracy of biomarkers in diagnosis of PC (Table 7, Fig. 1)

CEMIP demonstrated the highest diagnostic accuracy at cut off point > 3.59 ng/ml (84.3%) for PC compared to CA19-9 and CEA (62.9% and 73.4%, respectively). Combination was combined CEMIP/ CEA that had higher accuracy (90%) for detection of PC than others.

DeLong test revealed a statistically significant difference between AUCs of CEA vs. CEMIP and CA19-9 vs. CEMIP but no difference was found between CEMIP alone and its combination with CEA or CA19-9.

**Table 7:** Accuracy of biomarkers in diagnosis of pancreatic cancer.

Indices	CA19-9 (u/l)	CEA (ng/ml)	CEMIP (ng/ml)	CEMIP/CA19-9	CEMIP/CEA
Sensitivity	58%	80%	95%	85%	100%
Specificity	69%	65%	84%	85%	90%
PPV	70.4%	71.8%	95.2%	85%	91%
NPV	56.4%	74.4%	93%	85%	100%
Accuracy	62.9%	73.4%	92%	85%	90%
Cut off point	> 31	> 7	> 3.59		
AUC	0.80	0.80	0.86	0.84	0.87
*P* value	< 0.001	< 0.001	< 0.001	< 0.001	< 0.001

*P* value was significant if < 0.05. PC: pancreatic cancer; CEA: carcinoembryonic antigen; CA19-9: cancer antigen 19 − 9; CEMIP: cell migration inducing protein; PPV: positive predictive value; NPV: negative predictive value; AUC: area under curve.

**Fig. 1:**
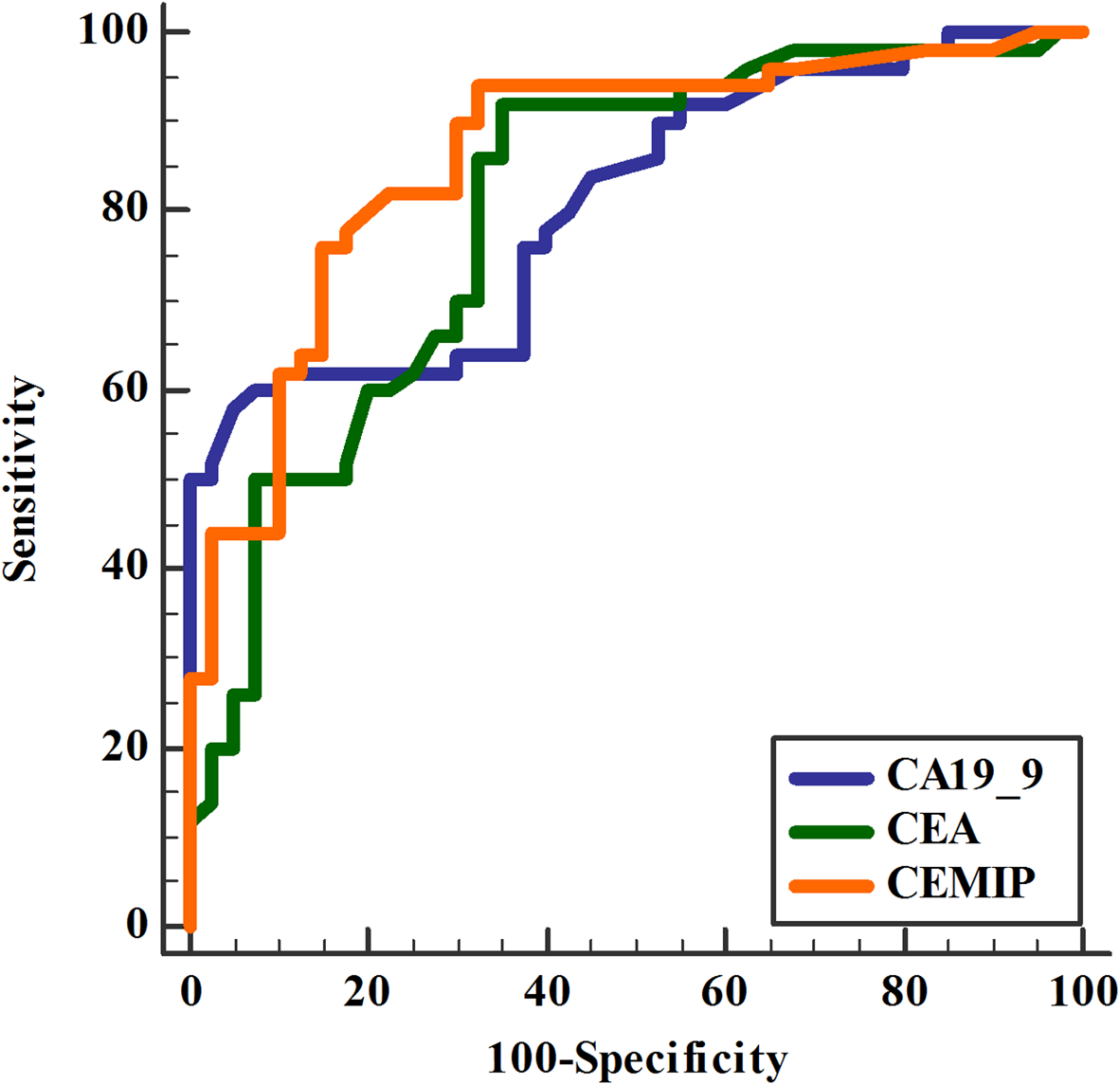
Accuracy of biomarkers in diagnosis of pancreatic cancer. CEA: carcinoembryonic antigen; CA19-9: cancer antigen 19 − 9; CEMIP: cell migration inducing protein.

### Accuracy of biomarkers in prediction the stage of PC (Table 8, Fig. 2)

CEMIP demonstrated the highest diagnostic accuracy (86.9%) for early PC compared to CA19-9 and CEA (59.1% and 68.4%, respectively). Combined CEMIP/ CEA that had higher 91% diagnostic accuracy compared to CEMIP alone.

DeLong test revealed a statistically significant difference between AUCs of CEA vs. CEMIP and CA19-9 vs. CEMIP but no difference was found between CEMIP alone and its combination with CA19-9. Meanwhile, combination of CEMIP/CEA was better than CEMIP alone.

**Table 8:** Accuracy of biomarkers in prediction the early stage of PC.

Indices	CA19-9 (u/l)	CEA (ng/ml)	CEMIP (ng/ml)	CEMIP/CA19-9	CEMIP/CEA
Sensitivity	60%	75%	90%	86%	93%
Specificity	58%	60%	83%	70%	89%
PPV	74.4%	70.5%	87%	80%	92%
NPV	71.9%	65.3%	86.7%	78.4%	90%
Accuracy	59.1%	68.4%	86.9%	79%	91%
Cut off point	> 28.90	> 6.01	> 2.33		
AUC	0.56	0.52	0.72	0.67	0.80
*P* value	0.41	0.85	0.004	< 0.001	< 0.001

*P* value was significant if < 0.05. PC: pancreatic cancer; CEA: carcinoembryonic antigen; CA19-9: cancer antigen 19 − 9; CEMIP: cell migration inducing protein; PPV: positive predictive value; NPV: negative predictive value; AUC: area under curve.

**Fig. 2:**
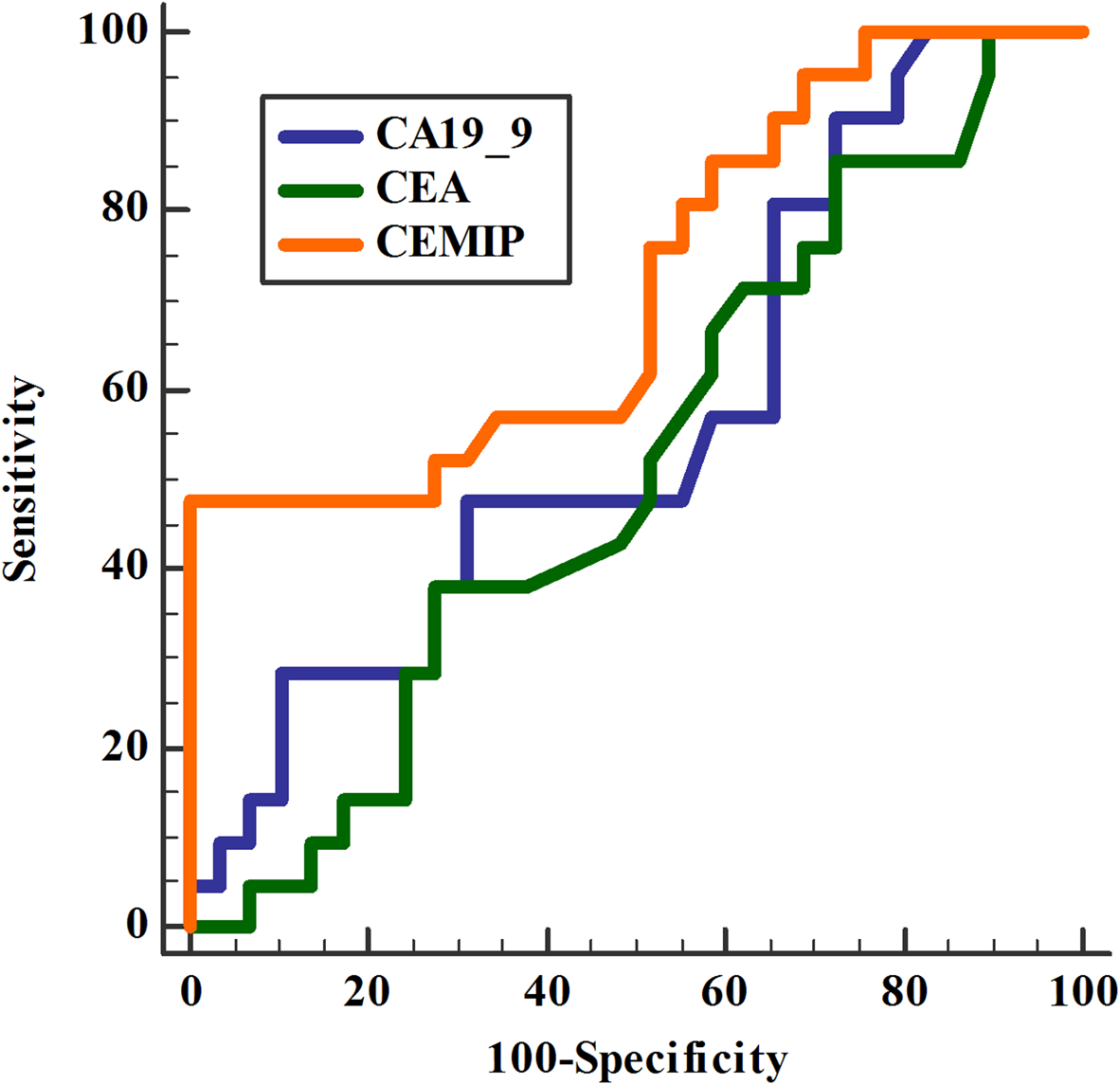
Accuracy of biomarkers in prediction the stage of pancreatic cancer. CEA: carcinoembryonic antigen; CA19-9: cancer antigen 19 − 9; CEMIP: cell migration inducing protein.

## Discussion

Several studies have explored the role of cell migration-inducing protein (CEMIP) in pancreatic cancer (PC), suggesting that CEMIP may be involved in early tumor detection, cancer cell migration, invasion, and is frequently associated with unfavorable prognosis across various malignancies^14–17^. In the current study we aimed to evaluate the clinical utility of serum CEMIP, CA19-9 and CEA in pancreatic cancer patients.

The principal findings of the current study indicated that patients in the pancreatic cancer (PC) group exhibited significantly elevated CEA levels compared to those with benign gastrointestinal tract (GIT) diseases and healthy controls. Furthermore, CEA levels were significantly higher in patients with late-stage PC compared to those with early-stage disease. In accordance with these findings, Meng, Shi^18^ said that PC group had significantly high serum CEA levels in compassion to the control group.

In line with the current study, Imaoka, Mizuno^19^ found that patients with metastatic PC had elevated CEA in comparison to those with early PC and the control group. In agreement with this findings, Kato, Kishiwada^20^ noticed that CEA was significantly elevated in non-operable pancreatic cancer in comparison to those with localized PC.

Additionally, a previous study reported a statistically significant difference in CEA levels based on lymph node metastasis, N grade, and T stage, with patients in the late-stage pancreatic cancer (PC) group exhibiting significantly higher values compared to those in the early-stage PC group^21^. This was consistent with many previous studies^22–24^.

A key finding in this study was that the pancreatic cancer (PC) group exhibited significantly higher CA19-9 levels compared to both the benign gastrointestinal (GIT) disease group and the control group. Moreover, CA19-9 levels increased with the progression of PC stages, with patients in the late-stage PC group showing significantly higher CA19-9 levels compared to those with early-stage PC.

In agreement with these findings; Kato, Kishiwada^20^ study corroborated that CA19-9 levels were significantly elevated in the pancreatic cancer (PC) group when compared to the control group. Furthermore, CA19-9 levels were found to increase with the progression of cancer stages, with markedly higher levels observed in stages III/IV compared to stages I/II. These results align with previous studies, which also reported elevated CA19-9 levels in PC patients relative to those with benign conditions. Additionally, a significant elevation in CA19-9 was noted in late-stage PC compared to early-stage disease^25^.

Another finding in the current study was that PC group had significantly higher CEMIP compared to benign GIT diseases and control group (p-value < 0.001) for both. But no significant difference was found between benign and control groups (p value = 0.13).

We found also, late-stage PC group had significantly higher CEMIP compared to early-stage PC. In line with the current study; previous meta-analysis of 11 literatures with 1355 patients were included. It was found that CEMIP was elevated in patients with PC^26^. Also, another study stated elevated CEMIP in patients with late PC^12^.

As regard correlation of CEMIP with other variables and markers in current study; we found that different markers had insignificant the correlation with other variables. Meanwhile, CEMIP had significant positive correlations with CEA (*r* = 0.65, *p* = 0.001) and CA19-9 (*r* = 0.37, *r* = 0.01). To our knowledge, no previous studies discussed the correlation between CEMIP with traditional biomarkers of PC (CA1-9 and CEA).

The primary findings of the current study indicated that, for the diagnosis of pancreatic cancer (PC), CEMIP demonstrated the highest diagnostic accuracy (92%) at a cutoff point of > 3.59 ng/ml, outperforming CEA, which exhibited an accuracy of 73.4% at a cutoff point of > 7 ng/ml, and CA19-9, which showed an accuracy of 62.9% at a cutoff point of > 31 ng/ml.

For the diagnosis of late-stage PC, CEMIP again exhibited the highest diagnostic accuracy (90.1%) at a cutoff point of > 5.70 ng/ml, compared to CEA, which had an accuracy of 72.2% at a cutoff point of > 9 ng/ml, and CA19-9, which demonstrated an accuracy of 66.3% at a cutoff point of > 34.56 ng/ml.

Lee, Jang^12^ compared CA19-9 alone to combined CA 19 − 9 and CEMIP in diagnosis of PC. Combination with CEMIP showed markedly improved AUROC over that of CA 19 − 9 alone in the diagnosis of pancreatic cancer against normal.

There are many previous studies that proved combination of CEA with CA19-9 had better diagnostic accuracy of PC and different stages of PC in comparison to each of CA19-9 and CEA alone^27–29^. van Manen, Groen^17^ noticed that combined CEA/CA19-9 had better diagnostic accuracy for PC (91.4%) in comparison to CEA (83.3%) and CA19-9 (73.6%)^17^.

The current study acknowledges certain limitations as small, single-center design, and lack of external validation. Also, we didn’t study its long-term effects as on survival, recurrence after surgery, response to chemotherapy. Lastly, potential assay variability for ELISA-based quantification. Yet, there were several strengths in the present study. First, this study is the first to investigate the role of CEMIP in pancreatic cancer in our locality. All clinical data and blood samples were collected from a perspective cohort of patients with pancreatic cancer.

## Conclusion

The analysis conducted in the present study demonstrated that CEMIP exhibited superior diagnostic performance in pancreatic cancer compared to CEA and CA19-9. This suggests that CEMIP levels may serve as a valuable biomarker for distinguishing patients with pancreatic cancer from healthy individuals.

Nonetheless, further multicenter studies with larger patient cohorts are warranted to validate these findings. Also, future studies should include other proteins involved in signaling pathways related to pancreatic cancer to obtain the most suitable biomarkers.

## Data Availability

Availability of data and materials: The datasets used and/or analyzed during the current study are available from the corresponding author on reasonable request.
