# Correction: Evaluation of CEMIP in diagnosis of pancreatic carcinoma in comparison with other traditional markers

**DOI:** 10.1038/s41598-025-25425-2

**Published:** 2025-10-27

**Authors:** Randa Ahmed El Zohne, Ahmad Kamel Mostafa Abo Zaid, Omnia Abd El Moneim, Ahmed Mohamed Soliman, Dina Mohamed Safwat, Soad A. Eltokhy

**Affiliations:** 1 Department of Clinical Pathology, Faculty of Medicine, Assiut University, Assiut, 17515 Egypt; 2 Department of General Surgery-Faculty of Medicine, Assiut University, Assiut, Egypt; 3 Clinical Pathology Department, National Cancer Institute, Cairo University, Cairo, Egypt

Correction to: *Scientific Reports* 10.1038/s41598-025-07911-9, published online 03 July 2025

The original version of this Article contained an error in Affiliation 3, which was incorrectly given as ‘Department of Clinical Pathology, National Cairo Institute, Cairo, Egypt’. The correct affiliation is listed below:

3. Clinical Pathology Department, National Cancer Institute, Cairo University, Cairo, Egypt.

The original Article has been corrected.

